# Effects of a 12-week aerobic exercise on markers of hypertension in men 

**DOI:** 10.15171/jcvtr.2018.26

**Published:** 2018-09-10

**Authors:** Behrouz Baghaiee, Pouran Karimi, Khadije Ebrahimi, Saeed Dabagh Nikoo kheslat, Mohammad Hossein Sadeghi Zali, Amir Mohammad Daneshian Moghaddam, Mohammad Sadaghian

**Affiliations:** ^1^Department of Physical Education and Sports Science, Jolfa Branch, Islamic Azad University, Jolfa, Iran; ^2^Neuroscience Research Center, Tabriz University of Medical Sciences, Tabriz, Iran; ^3^Department of Physical Education and Sports Science, Marand Branch, Islamic Azad University, Marand, Iran; ^4^Department of Exercise Physiology, Faculty of Sport Sciences and Physical Education, University of Tabriz, Tabriz, Iran; ^5^Department of Microbiology, Faculty of Veterinary Medicine, Urmia Branch, Islamic Azad University, Urmia, Iran; ^6^Department of Agriculture, Faculty of Veterinary Medicine, Shabestar Branch, Islamic Azad University, Shabestar, Iran; ^7^Department of Pathobiology, Faculty of Veterinary Medicine, Shabestar Branch, Islamic Azad University, Shabestar, Iran

**Keywords:** Hypertension, Exercise, Oxidative Stress, Lifestyle

## Abstract

***Introduction:*** This study was aimed at determining the effects of a 12-week aerobic exercise program on markers of hypertension in men.

***Methods:*** The study was of a semi-experimental design featuring repeated measurements. A total of 40 men (age range=37.9 ± 2.68) with primary hypertension were divided into two groups, namely, the exercise group (n=20) and the control group (n=20) (systolic blood pressure [SBP]: 140.531 ± 0.23, diastolic blood pressure [DBP]: 90.71 ± 0.05). The exercise group participated in a 12-week aerobic exercise program (55% to 70% of HRmax). Blood samples were taken from both groups at the baseline and at the 4th, 8th, and 12th weeks of the training program for the assessment of adiponectin, paraoxonase-1 (PON-1), and hydrogen peroxide (H2 O2 ) levels as the markers for investigation. A linear mixed model was also used to evaluate the association among the markers.

***Results:*** In the exercise group, exercise reduced the SBP and DBP at week 12 (*P*=0.031 and 0.023, respectively), and adiponectin increased at weeks 8 and 12 (*P*=0.014 and 0.001, respectively). The plasma PON-1 level showed a significant increase in all the three stages of measurement (*P*=0.007, 0.004, and 0.002 at weeks 4, 8, and 12, respectively), whereas the H2 O2 levels showed a significant decrease at weeks 8 and 12 (*P*=0.013 and 0.011, respectively). The control group exhibited significantly decreased PON-1 (*P*=0.003) and adiponectin (*P*=0.025) levels but significantly increased SBP at week 12 (*P*=0.032).

***Conclusion:*** The exercise-induced reduction of oxidative stress exerts a considerable effect on the reduction of blood pressure in hypertensive patients. According to our results increase in oxidative stress has the great impact on the of blood pressure.

## Introduction

Primary hypertension or high blood pressure (HBP) is a risk factor for cardiovascular disease in people of all ages and contributes significantly to mortality rates. HBP is a complex and polygenic medical condition with high prevalence among almost all populations. Its development is caused by many factors, but the most important are impairment in endothelial function and the renin–angiotensin system and hyperactivity in the sympathetic nervous system.^1-3^

Assessments of the relationship between body weight and blood pressure have shown that each 10 kg loss in weight and fat under the skin significantly reduces the risk of systolic and diastolic hypertension.^4,5^ Adipose tissue releases several hormones, including leptin, visfatin, tumor necrosis factor alpha, interleukin-6, and adiponectin.^6^ Among these hormones, adiponectin is positively associated with the regulation of glucose metabolism, increased sensitivity to insulin, beta-oxidation, and improved protection against cardiovascular diseases. Low levels of adiponectin are associated with the development of HBP, and decreased adiponectin has been observed in obese individuals. Reduced adiponectin is therefore a worrisome issue for people who are overweight or living a sedentary lifestyle.^6,7^

The development of HBP is associated with other factors, such as increased reactive oxygen species (ROS) and oxidative stress.^8,9^ ROS contribute to decreased vasodilation and hypertension through their reaction with nitric oxide (NO), their formation of peroxynitrite, and their impairment of endothelial activity.^10^ In obese people, high serum low-density lipoprotein (LDL) is expected to result in high levels of lipid peroxidation,^11^ which highlights the importance of paraoxonase-1 (PON-1). PON-1 is an enzyme that interacts with high-density lipoprotein (HDL) and LDL, thereby preventing them from being oxidized by ROSs.^12^ In this respect, PON-1 can be considered contributory to the regulation of blood pressure.^13^ Free radicals are likewise known as significant factors for the negative regulation of adiponectin,^14^ but the extent to which PON-1 variations may affect adiponectin levels is unclear given the antioxidant effects of PON-1.

Pursuing an active lifestyle and engaging in exercise can control blood pressure and levels of adiponectin, hydrogen peroxide (H_2_­O_2_), and PON-1. To date, studies on the long-term effects of adiponectin in middle-aged patients with HBP are scarce. Among the few efforts in this regard are those by Pasqualini et al^15^ and Parsian et al.^16^ Pasqualini et al probed into the effects of 8 weeks of moderate exercise on the adiponectin levels of 44-year-old patients suffering from hypertension and found increased adiponectin levels and improved arterial function.^15^ Parsian et al also reported increased levels of adiponectin in patients with type-2 diabetes after 8 weeks of physical activity.^16^ To the best of our knowledge, no study has delved into the association between adiponectin variations and PON-1 and H_2_­O_2_ in primary hypertensive patients and the effects of exercise on this relationship. This may explain the current ambiguity with respect to the effects of ROS and PON-1 on adiponectin variations, especially in patients with HBP. Furthermore, no well-established research on the effects of exercise on PON-1 in hypertensive patients can be found. Motivated by these deficiencies, we investigated the effects of a 12-week moderate aerobic exercise program on the relationship among changes in plasma PON-1, H_2_­O_2_, and adiponectin in 35- to 42-year-old inactive males suffering from HBP.

## Materials and Methods

This research was a semi-experimental study conducted in accordance with a repeated-measures design. The study population comprised 35- to 42-year-old hypertensive men living in Tabriz, Iran. After announcements made in 2015, 50 people volunteered to participate in the study. To determine their eligibility for inclusion, we asked the volunteers to complete medical history and physical activity questionnaires.^17,18^ After filling in consent and medical history forms, all the volunteers underwent a series of physiological measurements, which included a recording of their heart rates using an OPTIMA SE-315 (South Korea), systolic and diastolic blood pressure levels (SBP and DBP) using an OPTIMA SE-315 pressure set (South Korea), weight and height using a Seca stadiometer and scale (Germany), and body mass index (BMI) using a portable bioelectrical impedance system (Body Composition, Omron, China). To measure blood pressure and heart rate, subjects were asked to sit in the back seat for 30 minutes and did not use tea and coffee and smoking for 2 hours before. Purposive sampling was then conducted to select twenty 36- to 40-year-old volunteers who met the inclusion criteria. Individuals with a body mass index (BMI) greater than 26 kg.m², a heart rate above 82 bpm, an SBP greater than 140, a DBP greater than 90 mm Hg, and no history of regular exercise were selected for participation. Individuals with a history of regular exercise, who did not have the necessary characteristics for heart rate, SBP, DBP and BMI and with underlying illnesses such as heart failure were excluded from study.

The participants were then divided into two groups: the exercise group (Ex) (n=20) and the control group (Con) (n=20). All the subjects suffered from primary hypertension and had no history of taking medication for this condition. But during the study, the subjects were referred to a specialist physician and they took blood pressure medication under the supervision of a physician. The participants were also advised to avoid consuming caffeine, nicotine, alcohol and avoid exhaustive workouts (which can influence responses to blood pressure and stress) in the previous 48 hours.^17^

The basic physiological indices were re-measured, and venous blood samples were taken to evaluate the serum PON-1, H_2_O_2_, and adiponectin levels of the participants. The subjects in the exercise group participated in a 12-week program that involved aerobic exercises having an intensity of 55% to 70% of the maximum heart rate (HRmax).^17^ The subjects in the control group were told to refrain from participating in regular exercises during the study period. Changes in the physiological indices and the PON-1, H_2_O_2_, and adiponectin levels of all the subjects were measured at weeks 4, 8, and 12 (48 hours after the last exercise).

### Aerobic training protocol

Exercise sessions were carried out three times per week, with each session lasting for an average of 45 minutes. The initial sessions were performed at an HRmax of 50% for an average of 25 minutes, but after the subjects gained sufficient physical fitness, the sessions were intensified to an HRmax of 70% for a 45-minute duration. HRmax is a reliable index that performs better than do prediction equation methods for determining heart rate and exercise intensity.^17^ Exercises were supervised by Exercise Physiology Specialist.

### Adiponectin

Plasma adiponectin was measured by ELISA (enzyme-linked immunosorbent assay) using adiponectin kit (Adipogen.co-South Korea) with a sensitivity of 0.1 μg/mL.

### PON-1

PON-1 activity was measured using paraoxon substrate (Sigma Chemical Co.). Serum (20 μL) was added to Tris/HCl buffer (100 mmol, PH=8) containing 2 mM of paraoxon and 2 mM of calcium chloride (CaCl_2_). The paraoxon hydrolysis rate was measured using a spectrophotometer at a wavelength of 412 nm and with the release of paranitrophenol at 37°C. Enzyme activity was calculated using an extinction coefficient of 18290 mol/L and expressed in nmol/min/mL serum.

### H_2_O_2_

To measure H_2_O_2_ by the FOX-1 method, 100 μmol of FOX-1 reagents (xylenol orange), 250 μmol of ammonium ferrous sulfate, 100 μmol of sorbitol, and 25 μmol of sulfuric acids were prepared. A 1.5 mL Eppendorf microtube was used to mix 50 mL of the sample with 950 mL of prepared FOX-1 reagent in a vortex machine. The mixture was incubated for 30 minutes at 40°C (carbon dioxide incubator). Sample absorbance was recorded using a spectrophotometer at a wavelength of 650 nm.

### Statistical analysis

The Kolmogorov–Smirnov test was performed to verify the normal distribution of data, and the repeated-measures Bonferroni test was carried out to compare the blood indices measured at different stages and their base values. A linear mixed model was also used to evaluate the relationship among the indices. All the data were analyzed using the Statistical Package for the Social Sciences version 22 (SPSS, Chicago, IL, USA). In all the analyses, an α ≤ 0.05 was considered statistically significant.

## Results

### Exercise group

The exercise group showed a statistically significant weight loss at week 12 (*P *= 0.03). In this group, moderate-intensity exercise decreased the SBP and DBP, but only the reduction measured at week 12 was high enough to be considered significant (*P *= 0.031 and 0.023, respectively). The resting heart rates of the group significantly decreased at weeks 8 and 12 (both *P *= 0.001) (All the measured data were compared with their base values.) (Tables 1 and 2).

**Table 1:** Physiological and demographic characters at different stages of research

**Markers**	**Group**	**Research stage**				***P*** ^*^ ** (Intra group)**	
		**Basal**	**Week 4**	**Week 8**	**Week 12**	**Ex**	**Con**
Age (y)	Ex	37.9±2.68	37.9±2.68	37.9±2.68	37.9±2.68	-	-
	Con	38.3±2.17	38.3±2.17	38.3±2.17	38.3±2.17		
Height (cm)	Ex	176.11±1.23	176.11±1.23	176.11±1.23	176.11±1.23	-	-
	Con	175.09±2.14	175.09±2.14	175.09±2.14	175.09±2.14		
Weight (kg)	Ex	83.21±3.21	82.32±2.11	80.43±1.01	79.23±1.22	*P*_1_=0.658 *P*_2_=.0.51 *P*_3_=0.03	*P*_1_=0.688 *P*_2_=.0.109 *P*_3_=0.048
	Con	83.18±5.11	83.23±5.13	85.32±4.14	86.34±3.18		
BMI (kg/m^2^)	Ex	26.84±2.14	26.55±2.11	25.94±1.01	25.55±1.22	*P*_1_=0.706 *P*_2_=.0.061 *P*_3_=0.043	*P*_1_=0.891 *P*_2_=.0.509 *P*_3_=0.077
	Con	27.18±1.27	27.19±1.28	27.88±2.5	28.21±3		
Rest heart rate (beats/min)	Ex	85.5±2.66	84.4±1.77	82.9±2.02	80.5±1.5	*P*_1_=0.146 *P*_2_=.0.016 *P*_3_=0.001	*P*_1_=0.478 *P*_2_=.0.221 *P*_3_=0.112
	Con	88 ±3.19	87±2.53	86.5±2.41	86.2±2.61		

* Based on Repeated measure and Bonferroni test (*P* ≤ 0.05), *P*_1_: Comparison of fourth week with Basal, *P*_2_: Comparison of the eighth week with Basal, *P*_3_: Comparison of the twelfth week with Basal

**Table 2:** Systolic and Diastolic blood pressure at different stages of research

**Markers**	**Group**	**Research stage**				***P*** ^*^	
		**Basal**	**Week 4**	**Week 8**	**Week 12**	**Ex**	**Con**
Systolic blood pressure (mm Hg)	Ex	140.53±0.23	140.5±0.22	140.47±0.24	139.97±0.2	*P*_1_=0.555 *P*_2_=.0.534 *P*_3_=0.031	*P*_1_=0.999 *P*_2_=.0.326 *P*_3_=0.032
	Con	140.54±0.22	140.54±0.21	140.55±0.21	140.58±0.21		
Diastolic blood pressure (mm Hg)	Ex	90.71±0.5	90.707±0.34	90.704±0.4	90.701±0.25	*P*_1_=0.478 *P*_2_=.0.143 *P*_3_=0.023	*P*_1_=0.999 *P*_2_=.0.623 *P*_3_=0.057
	Con	90.7±0.04	90.71±0.03	90.71±0.05	90.72±0.04		

* Based on Repeated measure and Bonferroni test (*P* ≤ 0.05), *P*_1_: Comparison of fourth week with Basal, *P*_2_: Comparison of the eighth week with Basal, *P*_3_: Comparison of the twelfth week with Basal.

**Figure 1:**
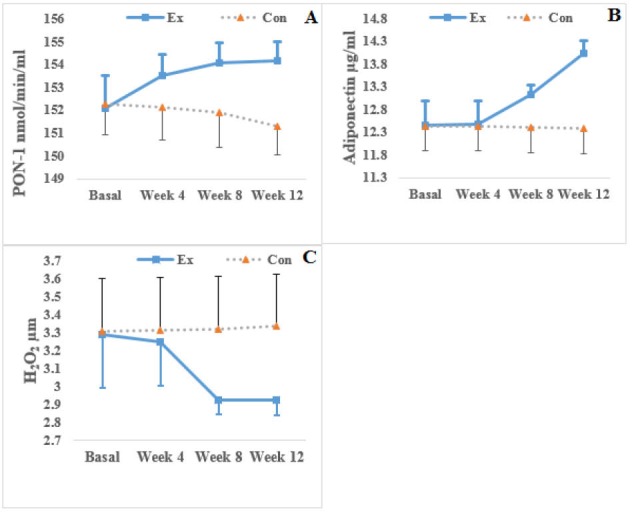
Level of PON-1, adiponectin and H2O2 in exercise and control groups.

The measurements made for the exercise group showed an increase in adiponectin levels as the result of moderate-intensity exercise, and this increase was significant at weeks 8 and 12 (*P *= 0.014 and 0.001, respectively). The plasma PON-1 levels of the group significantly increased at weeks 4, 8, and 12 (*P *= 0.007, 0.004, and 0.002 respectively), but their H_2_O_2_ levels significantly decreased only at weeks 8 and 12 (*P *= 0.013 and 0.011, respectively) (Figure 1).

The exercise group showed a significant relationship between adiponectin and H_2_O_2_ and PON-1 (*P *≤ 0.05), but the relationship between the last two markers was not significant. The SBP and DBP of the group was not significantly correlated with either adiponectin or PON-1. However, the relationship between the SBP and DBP and H_2_O_2_ was significant (*P *= 0.002 and 0.048, respectively) (Table 3).

**Table 3:** The relationship between markers by using linear mixed model

**Marker-1**	**Maker-2**	**Group**	***P*** ^*^
Paraoxonase-1	Adiponectin	Ex	0.001
		Con	0.089
Hydrogen Peroxide	Adiponectin	Ex	0.003
		Con	0.478
Paraoxonase-1	Hydrogen Peroxide	Ex	0.389
		Con	0.001
Adiponectin	SBP	Ex	0.15
		Con	0.633
Adiponectin	DBP	Ex	0.651
		Con	0.211
Hydrogen Peroxide	SBP	Ex	0.02
		Con	0.001
Hydrogen Peroxide	DBP	Ex	0.048
		Con	0.01
Paraoxonase-1	SBP	Ex	0.165
		Con	0.001
Paraoxonase-1	DBP	Ex	0.52
		Con	0.51

* Based on mixed model statically analyze (*P* ≤ 0.05)

### Control group

The control group exhibited significantly decreased PON-1 (*P *= 0.003) and adiponectin (*P *= 0.025) at week 12 (Figure 1). However, the group exhibited a significant increase in their SBP and weights during this week (*P*=0.032 and 0.048, respectively) (Tables 1 and 2). The results showed a significant relationship between PON-1 and H_2_O_2_ (*P *= 0.001) and between SBP and DBP and H_2_O_2_ (both *P *= 0.001). A significant relationship was also observed between the PON-1 levels and SBP of the group (*P *= 0.001) (Table 3).

## Discussion

As previously indicated, this study was aimed at assessing the relationship between the changes in PON-1, H_2_O_2_, and plasma adiponectin and the changes in SBP and DBP in hypertensive men during and after 12 weeks of moderate-intensity exercise. Our assessments showed that this type of exercise reduced the blood pressure of the hypertensive males but that such reduction was not significant at weeks 4 and 8. A significant reduction was achieved only at the end of the program (week 12). These findings indicated that controlling blood pressure in hypertensive patients requires long-term commitment to exercise—a conclusion also reached by Sherman et al.^19^ A more important point, however, is the identification of the mechanisms through which exercise reduces blood pressure in hypertensive individuals. The present study examined three major markers of HBP, namely, PON-1, H_2_O_2_, and adiponectin. Adiponectin is a protein that has received much attention in the vascular health research conducted in the past decade. Its concentration can be affected by changes in body weight and extent of physical activity.^20,21^ Evidence also suggested that adiponectin decreases with weight gain and physical activity reduction.^22,23^ We likewise found a correlation between weight loss and a significant increase in adiponectin, as evidenced by the increased levels of this protein at weeks 8 and 12 in the exercise group.

The findings of previous studies have shown that increased levels of H_2_O_2_, angiotensin II, and inflammatory cytokines somehow contribute to the reduction of adiponectin.^24,25^ Although not considered a free radical, H_2_O_2_ is categorized as an ROS and plays a direct and effective role in increasing oxidative stress and free radicals.^26^ In the present study, exercise significantly decreased H_2_O_2_ levels at weeks 8 and 12. Of relevance, as well, is the relationship between the changes H_2_O_2_ and adiponectin. Our findings revealed that under the influence of aerobic exercise, a reduction in H_2_O_2_ increased adiponectin levels, reflecting a significant relationship between these markers. On the basis of these findings, reduced oxidative stress—a direct effect observed in this work—is one of the most influential factors for the increase in adiponectin, even in people with HBP.

The role of PON-1 in this process cannot be disregarded. PON-1 is an enzyme that is expressed in the liver; it is associated with HDL and LDL and contributes to the reduced oxidation of blood fats and reduced oxidative stress.^27^ Although the concentration of this enzyme can also be affected by weight gain and lack of physical activity,^28^ whether exercise increases PON-1 levels has not been confirmed.^29^ Our statistical analysis showed that moderate exercise significantly increased PON-1 levels at weeks 4, 8, and 12 and suggested that changes in PON-1 affected adiponectin levels. These effects, along with the increase in PON-1, elevated the adiponectin levels of the subjects.

Although one might think that PON-1 triggers the above-said effect through reducing H_2_O_2_, our findings showed no significant relationship between changes in PON-1 and H_2_O_2_, and the increase in PON-1 only led to a negligible reduction in H_2_O_2_. So the more probable scenario is the involvement of other mechanisms, such as the role of PON-1 in the reducing anti-inflammatory and pro-inflammatory cytokines.^30^ Reports on the role of inflammatory cytokines in the increase of oxidative stress are available.^31^ But it should also be noted that for hypertensive individuals, increased PON-1 cannot be confidently acknowledged as the primary factor for reduced H_2_O_2_, as increased gene expression and activity of antioxidant enzymes (including SOD) is also effective in this respect.^32^ However, another limitation of this study was the lack of measurement of these enzymes.

Although 12 weeks of physical activity changed the study variables (i.e., PON-1, adiponectin, and H_2_O_2_ levels), the mechanism through which physical activity changes blood pressure through these variables should be accurately determined. Increased adiponectin has been suggested as contributory to reduced blood pressure, and some studies have reported that adiponectin increases NO.^33^ Researchers believe that adiponectin plays an important role in the increased activity of adenosine monophosphate-activated protein kinase (AMPK) in endothelial cells through adiponectin receptor 1 and adiponectin receptor 2.^34^ Through AMPK, therefore, adiponectin contributes to the activation and phosphorylation of endothelial nitric oxide synthase (eNOS).^35^ Evidence also suggested that heat shock protein 90 (Hsp90) plays an important role in eNOS regulation, and reports indicated that adiponectin contributes to NO production by activating Hsp90.^34,36^ NO is one of the factors that affect vascular dilation, and various studies have reported the positive effects of exercise on increasing eNOS and NO.^37,38^ Nevertheless, our assessments showed no significant relationship between the increased adiponectin levels of the subjects and their reduced SBP and DBP. Despite the increase in adiponectin as a result of moderate exercise, its role in regulating the blood pressure of hypertensive individuals was not significant. Given the lack of adequate background, we cannot claim a certain cause for this result, but reduced levels of adiponectin receptors can act as an important factor, as indicated in some studies in which a reduced level of adiponectin receptors in the endothelium of hypertensive individuals was reported.^39^

The role of H_2_O_2_ is also worthy of attention. Our results showed a significant relationship between SBP and DBP and decreased H_2_O_2_. Some researchers argued that a reduction in H_2_O_2_ contributes to the reduction in blood pressure probably through increased adiponectin and PON-1.^40,41^ Given that our results suggested no such connection, focus should be shifted toward other mechanisms. Researchers contended that through interaction with eNOS and NO activities, ROSs contribute to the production of ON-, which is a ROS that can react with other molecules.^8^ Therefore, interference in the activity of eNOS and reduced levels of NO are implicated in increased blood pressure. Evidence suggested that ROSs contribute to the reduction of tetrahydrobiopterin, which is one of the cofactors involved in the production of NO.^42^ Nevertheless, some results implied that ROSs play a role in the increase in growth factors and the activation of matrix metalloproteinases, which both contribute to increased blood pressure.^43,44^ Correspondingly, a reduction in H_2_O_2_ can probably regulate SBP and DBP by reducing the aforementioned mechanisms.

The findings pertaining to the control group subjects, who were asked to refrain from participating in regular exercise, showed a significant relationship between increased H_2_O_2_ and increased SBP and DBP. The increased H_2_O_2_ in the control group is ascribed primarily to the reduction in PON-1. The results also indicated a significant correlation between the reduction in PON-1 and increased SBP.

## Conclusion

Overall, the findings showed that for the middle-aged hypertensive men, 12 weeks of moderate-intensity aerobic exercise reduced H_2_O_2_ and increased PON-1 and adiponectin levels. However, the statistical assessments suggested that the exercise-induced reduction of oxidative stress indices exerted the greatest effects on the decreased blood pressure levels of the subjects. Similar results were obtained for the controls; increased oxidative stress indices caused by lack of physical activity had the strongest effect on increased blood pressure.

The limitations of this study are worth noting. Our results regarding the association among moderate exercise, PON-1 level, and adiponectin level should be interpreted with caution as the research limitations presented difficulties in confirming the exact mechanism through which PON-1 variations increase adiponectin. Although PON-1 may be argued as triggering the aforementioned effect by reducing H_2_O_2_, our findings showed no significant relationship between changes in PON-1 and H_2_O_2_. Additionally, the increase in PON-1 led to a negligible reduction in H_2_O_2_. A more probable scenario is the involvement of other mechanisms, such as the role of PON-1 in reducing anti-inflammatory and pro-inflammatory cytokines.^30^ Reports on the role of inflammatory cytokines in increased oxidative stress are available.^31^ Note that for hypertensive individuals, increased PON-1 cannot be confidently acknowledged as the primary factor for reduced H_2_O_2_ because increased gene expression and antioxidant enzyme activity (including superoxide dismutase) are also implicated in the reduction of H_2_O_2_.^32^ The lack of measurement of these enzymes and the lack of evaluation for angiotensin II levels are the other limitations of our work. These parameters should be included in future studies.

## Ethical approval

This study was approved by the Ethics Committee of the Islamic Azad University Jolfa Branch.

## Competing interests

All authors declare no competing financial interests exist.

## Acknowledgments

We would like thanks Islamic Azad University Jolfa Branch for their support and also all the participants in this study.
